# A Rapid Non-Destructive Hyperspectral Imaging Data Model for the Prediction of Pungent Constituents in Dried Ginger

**DOI:** 10.3390/foods11050649

**Published:** 2022-02-23

**Authors:** Nahidul Hoque Samrat, Joel B. Johnson, Simon White, Mani Naiker, Philip Brown

**Affiliations:** 1 School of Health, Medical and Applied Sciences, Central Queensland University, Bundaberg, QLD 4670, Australia; s.c.white@cqu.edu.au (S.W.); p.h.brown@cqu.edu.au (P.B.); 2 Institute for Future Farming Systems, Central Queensland University, Bundaberg, QLD 4670, Australia; 3 School of Health, Medical and Applied Sciences, Central Queensland University, Rockhampton, QLD 4701, Australia; j.johnson2@cqu.edu.au (J.B.J.); m.naiker@cqu.edu.au (M.N.); 4 Institute for Future Farming Systems, Central Queensland University, Rockhampton, QLD 4701, Australia

**Keywords:** hyperspectral imaging, non-destructive detection, ginger, gingerols, shogaols

## Abstract

Ginger is best known for its aromatic odour, spicy flavour and health-benefiting properties. Its flavour is derived primarily from two compound classes (gingerols and shogaols), with the overall quality of the product depending on the interaction between these compounds. Consequently, a robust method for determining the ratio of these compounds would be beneficial for quality control purposes. This study investigated the feasibility of using hyperspectral imaging to rapidly determine the ratio of 6-gingerol to 6-shogoal in dried ginger powder. Furthermore, the performance of several pre-processing methods and two multivariate models was explored. The best-performing models used partial least squares regression (PSLR) and least absolute shrinkage and selection operator (LASSO), using multiplicative scatter correction (MSC) and second derivative Savitzky–Golay (2D-SG) pre-processing. Using the full range of wavelengths (~400–1000 nm), the performance was similar for PLSR (R^2^ ≥ 0.73, RMSE ≤ 0.29, and RPD ≥ 1.92) and LASSO models (R^2^ ≥ 0.73, RMSE ≤ 0.29, and RPD ≥ 1.94). These results suggest that hyperspectral imaging combined with chemometric modelling may potentially be used as a rapid, non-destructive method for the prediction of gingerol-to-shogaol ratios in powdered ginger samples.

## 1. Introduction

The ginger plant (*Zingiber officinale* Roscoe) possesses an edible rhizome best known for its pungent flavour. It also possesses beneficial medicinal properties, including antioxidant activity [1], anti-inflammatory action [2], pro-cardiovascular health activity [3,4], and analgesic activity [5]. The worldwide production of ginger is approximately 800,000 tons/year, with India and China being the major producers [6]. The major exporters are China, Thailand, and Indonesia, who export to the United States, the United Kingdom, Saudi Arabia, and Japan [6].

**Figure 1:**
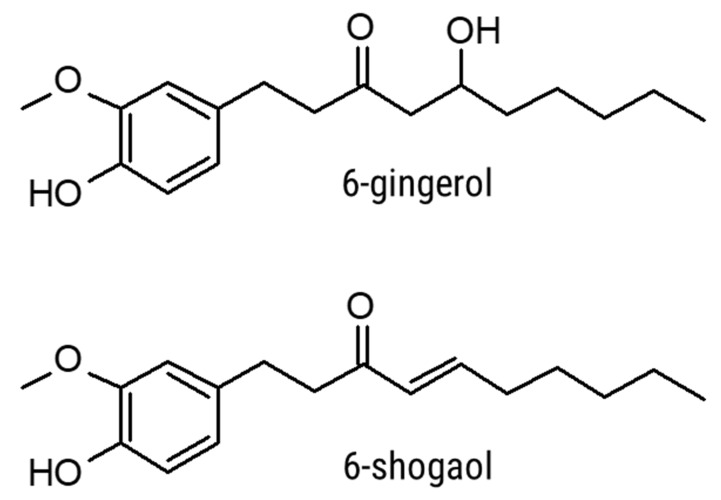
The structures of 6-gingerol and 6-shogaol.

The key bioactive compounds found in ginger—responsible for its pungent flavour and bioactive properties—are gingerols and gingerol derivatives [7]. These O-methoxyphenyl alkyl ketones are synthesised in the ginger rhizome through a combination of the phenylpropanoid and lignin biosynthesis pathways [8]. The most abundant pungent constituent present in fresh ginger is 6-gingerol, which possesses an alkyl side chain length of 6 carbons [9]. Under heat treatment, 6-gingerol undergoes an elimination dehydration reaction to form 6-shogaol [10], which is more pungent than 6-gingerol [11] and displays greater bioactivity [12]. The structures of these compounds are shown in Figure 1.

The levels of 6-gingerol and 6-shogaol in ginger samples vary according to the variety, growing conditions and any subsequent processing methods [9,13,14,15,16]. As suggested in [10], the ratio of 6-gingerol to 6-shogaol may be used as an indicator of ginger quality. Given the importance of these compounds and their derivatives in determining the sensory profile and hence the overall quality of ginger, routine measurement of these compounds would be greatly beneficial for quality assurance purposes. However, the concentrations of 6-gingerol and 6-shogaol are typically determined through expensive, time-consuming, liquid chromatography-based methods such as high-performance liquid chromatography (HPLC) or liquid chromatography-mass spectrometry (LC-MS) [9,17,18]. These methods are not suited to routine, in-line quality analyses of ginger products; consequently, there is increasing interest in developing rapid analytical techniques for assessing ginger quality. 

One method of rapid food quality analysis that is gaining attention is hyperspectral imaging (HSI) [19,20,21]. HSI is an image-based technique that uses a set of narrow-band images captured sequentially or simultaneously over an object within a specified spectral range (typically encompassing the visible and near-infrared regions). It provides both spatial and spectral information [22] and enables the identification and characterisation of chemical compounds in biological and food objects.

Due to the large amount of data generated through hyperspectral techniques, it is necessary to use appropriate chemometric tools to interpret the information contained in the spectra across the image [23,24]. These often include advanced algorithms for identifying key wavelengths that contribute to the predictive ability of the model, such as competitive adaptive reweighted sampling (CARS) [25], combined with iterative selection of successive projections algorithm (ISSPA) or a successive projections algorithm (SPA) [23,25]. The goal of these data processing techniques is to minimise contributions from the imaging instrument that do not relate to the composition of the sample being analysed, while maximising the impact of wavelengths which correspond to the analyte of interest [26]. 

Although HSI systems for the assessment of food and crop quality have used wavelengths between 350 and 1700 nm, the most commonly applied wavelengths are between 400 nm and 1000 nm [27,28,29,30,31]. Hyperspectral imaging has been successfully applied for the quality and safety assessment of horticultural products including mango, cucumber, almond, kiwifruit and banana [27,29,30,31,32], as well as other foodstuffs [28,33]. Furthermore, hyperspectral imaging has recently been used to measure the amount and distribution of moisture in ginger slices [34,35,36]. However, there are currently no known applications of HSI to assess pungent constituents in ginger. Investigations in [37] recently reported using Fourier-transform near-infrared spectroscopy to estimate the contents of zingerone, 6-gingerol, 8-gingerol, 10-gingerol, and 6-shogaol in dried, powdered ginger samples from China. As HSI also includes wavelengths in the near-infrared region, this suggests that HSI imaging systems may also be able to measure pungent constituents in ginger. Based on this hypothesis, this study aimed to investigate the feasibility of applying hyperspectral imaging to estimate the ratio of the two major pungent components, 6-gingerol and 6-shogaol, in dried ginger powder. 

This work is the first to demonstrate the prediction of bioactive compounds in ginger through hyperspectral imaging. This ability could be greatly beneficial for implementing real-time quality analysis of ginger products into the manufacturing process, through the use of HSI systems. Furthermore, it is also the first study to predict the gingerol-to-shogaol ratio of ginger samples using Vis-NIR wavelengths. Finally, the pre-processing methods are optimised for two regression methods (PLSR and LASSO), providing important information for future researchers of this topic. 

## 2. Data and Methods

### 2.1. Sample Collection, Preparation, and Chemical Analysis

A total of 89 samples of dried, processing-grade ginger powder of varying quality were sourced from Queensland ginger growers during 2018 and 2019. Samples were kept in an air-conditioned storage room (approx. 20 °C) prior to analysis. 

The gingerols and shogaols were extracted using protocols previously reported for this purpose [38,39,40]. Approximately 0.5 g of powdered ginger sample was suspended in 7 mL of 90% aqueous methanol and mixed end-over-end for 60 min. Following centrifugation (1000× *g*; 10 min), the supernatant was collected and the pellet extracted again with 7 mL of 90% methanol and mixed end-over-end for 20 min. The combined supernatants were made up to 15 mL with 90% methanol and syringe filtered (LabServ 0.2 µm nylon membrane) prior to direct HPLC analysis. Extractions and HPLC analyses were performed on each of the 89 samples in duplicate. The mean value obtained was used in subsequent statistical analyses and modelling. 

**Figure 2:**
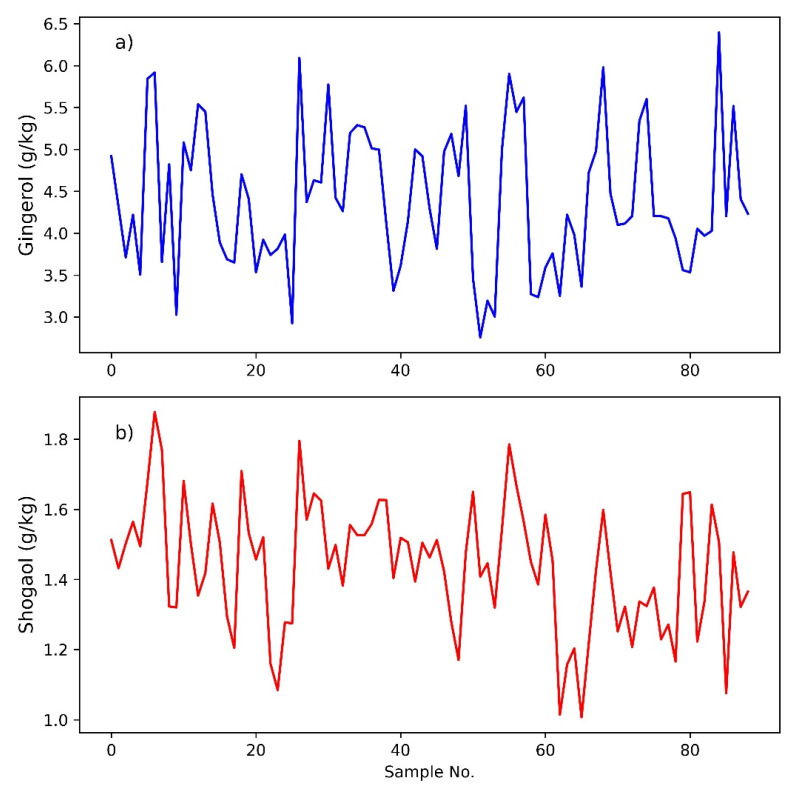
The concentration of 6-gingerol (**a**) and 6-shogaol (**b**) in the ginger samples, as measured by high-performance liquid chromatography analysis.

The HPLC method for gingerol and shogaol profiling was based on the method described in [9], but used a methanol gradient instead of acetonitrile. Separation and quantification of 6-gingerol and 6-shogaol were achieved on an Agilent 1100 HPLC system, comprising a G1313A autosampler, G1322A vacuum degasser, G1311A quaternary pump, G1316A thermostatted column compartment and G1365B multi-wavelength detector module. A reversed-phase C_18_ column was used (Agilent Eclipse XDB-C18; 150 × 4.6 mm; 5 µm pore size) with the column temperature controlled at 27 ± 0.8 °C. The injection volume was 5 µL, with gingerols and their derivatives detected and quantified at a wavelength of 230 nm. The mobile phase comprised of water and methanol at a flow rate of 1 mL/min, with a gradient beginning at 30% methanol (0 min), ramping to reach 60% methanol at 2 min, 63% at 10 min, 65% at 16 min and finally 100% at 28 min. The total run time was 33 min, with a post-run flush of 5 min. Quantification of 6-gingerol and 6-shogaol was achieved using an external calibration curve between 10 and 100 ppm, using authentic standards (Sigma-Aldrich, Sydney, Australia). The HPLC data for 6-gingerol and 6-shogaol contents are shown in Figure 2a,b.

### 2.2. Hyperspectral Imaging System and Acquisition

**Figure 3:**
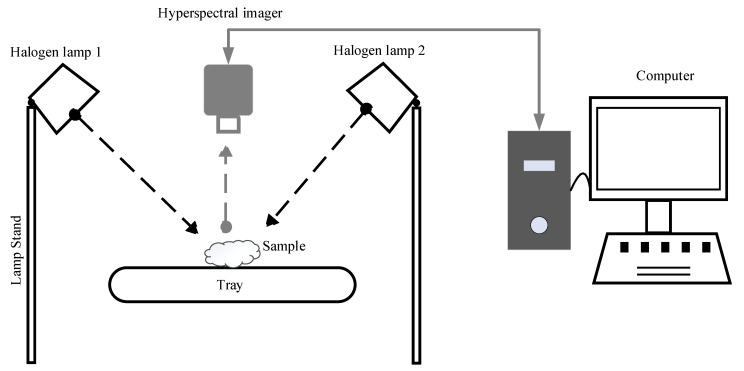
Schematic diagram of the hyperspectral imaging system in the laboratory.

Each of the 89 samples of dried ginger powder was scanned using a Specim IQ hyperspectral VS-NIR camera (Specim, Spectral Imaging Ltd., Oulu, Finland) arranged with two symmetrically placed 750 W tungsten halogen lamps (ARRILITE 750 Plus, ARRI, Germany) and a white reference tile (Figure 3). The camera employs a reflectance geometry and operates over the range 400–1000 nm with a spectral resolution of ~7 nm, providing a total of 204 wavebands. Consequently, each hyperspectral image is a volumetric image cube that stores both spatial and spectral information of the sample in a three-dimensional matrix.

### 2.3. Image Correction and Spectrum Extraction

The hyperspectral images were calibrated by a white reference and black reference image to remove the camera sensor’s dark current influence [30]. The following equation was used to calibrate the original hyperspectral images:

(1) $$I_{c}=\frac{I_{\mathrm{raw}}-I_{\mathrm{dark}}}{I_{\mathrm{white}}-I_{\mathrm{dark}}}$$

where I_raw_ is the original hyperspectral image, I_white_ is the white reference image, and I_dark_ is the dark reference image. The white image was captured from a white ceramic tile with 99% reflectance, while the dark image was automatically recorded inside the Specim sensor as part of the data recording workflow. The calibrated images were cropped by defining a square region (region of interest, ROI) over the ginger powder sample (see Figure S1 in the Supplementary File).

### 2.4. Spectral Pre-Processing

The hyperspectral datacube contained the influence of unwanted effects and noises (e.g., baseline shifts, light scattering, an uncontrolled external factor, and random noises) which could influence the results. To minimise these undesired effects and noises, spectral pre-processing was employed prior to the multivariate model development. The average spectrum was calculated from all pixels within the ROI to assist in averaging out the impact of random noise across this region. This average spectrum was used for subsequent processing.

As it is recommended that several types of pre-processing be used when developing multivariate calibration models [41], a number of pre-processing methods were trialled in this study, including standard normal variate (SNV), multiplicative scatter correction (MSC), mean filter with 5 × 5 window size (MF5), mean filter with 9 × 9 window size (MF9), and the first and second derivatives calculated using Savitzky-Golay smoothing (1D and 2D SG) [37,41,42]. The performance of each model created was evaluated in order to determine the optimal pre-processing method for the prediction of the ratio of 6-gingerol and 6-shogaol in dried ginger powder.

### 2.5. Predictive Modeling and Validation

**Figure 4:**
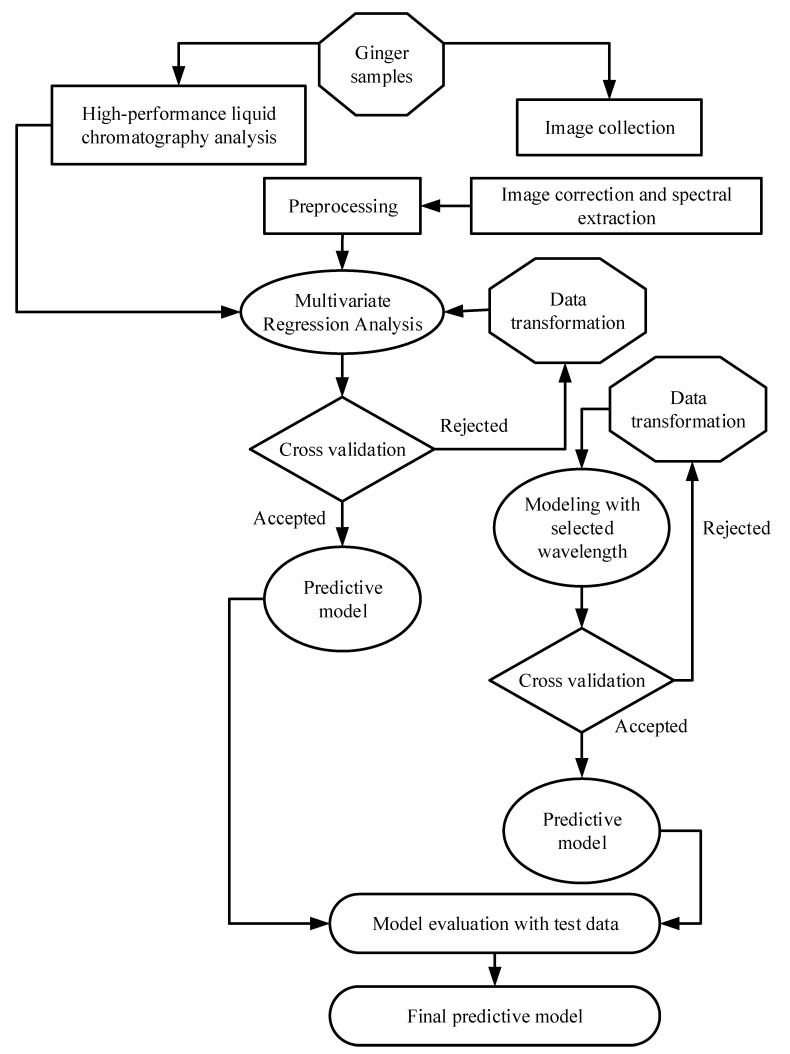
A schematic workflow of data processing and model development.

Two different multivariate regression approaches were used in this study. Partial least squares regression (PLSR) was chosen for its excellent performance and broad use in previous studies [34,43,44,45,46,47]. The second method, least absolute shrinkage and selection operator (LASSO) regression, was selected for its power to handle multiple collinear features [48]. Although no previous literature was found utilising LASSO regression for the HSI analysis of horticultural or food products, other authors have reported similar performance between LASSO regression and PLSR when working with satellite data [48]. All models built in this study were implemented in Python (version 3.6) using the models from the scikit-learn library. The model-building workflow is depicted in Figure 4.

#### 2.5.1. Partial Least Squares Regression

Partial least squares regression (PLSR) is a supervised and linear method of regression modelling that enables the exploration of the relationship between several dependent and independent variables. PLSR is the most widely used multivariate chemometric method. It has proved to be a reliable, robust, and accurate method in spectral data analysis [34,44,45]. The ratio of gingerol and shogaol from each sample correlated with their corresponding HSI spectral reflectance using the PLSR model. The PLSR is a multivariate projection algorithm used to model the relationship between independent variables matrix (X) and dependent variables (Y). This algorithm finds a set of latent variables (LV) in X to estimate the Y. The PLSR algorithm creates a linear model by decomposing values of both X (N × K) and Y (N × M) using the following relations:

(2) $$X={\mathrm{TP}}^{T}+E$$

(3) $$Y={\mathrm{TQ}}^{T}+F$$

where N is the number of observations, M is the number of Y variables, K is the number of X variables, P (K × J) is the matrix of X loadings, T (N × J) is the matrix of X scores, Q (J × M) is the loading vector of Y, E (N × K) and F (N × M) are error matrices, and J is the number of latent variables (LVs).

Both X and Y are expected to be partially modelled by the same LVs. The scores T are computed by X variables and weight matrix (K × J) linear combination. The value of the weight matrix is calculated to ensure maximising the covariance between the scores and the responses. The regression coefficient (β) is calculated from the W resulting from the model and optimum LVs. The PLSR algorithm is summarised in the following relations:

(4) y=βX+F

where y is the predicted Y variable.

Before the development of the PLSR model, the ratio of 6-gingerol to 6-shogaol was calculated from the quantitative HPLC results for each sample. The ratio of these two compounds was then predicted from the corresponding HSI spectra (204 wavelengths) using the proposed PLSR model. The dataset was randomly divided into training (80%) and test (20%) sets prior to model development. Data were divided into training and testing sets using the ‘train_test_split’ function from the sklearn.model_selection python package, based on a value of 20 for the ‘random_state’ used in train_test_split function. The training dataset was used to develop the model and the test dataset was used to determine how well the developed model could predict the gingerol–shogaol ratio in samples that were not included in model development. The full cross-validation (leave-one-out) method was used to determine the number of LVs, in order to obtain the best performance and avoid overfitting [49,50,51,52]. The optimal number of LVs was selected from the minimum predicted root mean square error (RMSE) obtained from the cross-validation set [53].

#### 2.5.2. LASSO Regression

LASSO is a regularisation technique based on an extension to linear regression and reduces model overfitting by balancing the model bias–variance trade-off using shrinking of the coefficients [48,54]. This technique can shrink coefficients to be precisely 0 and subsequently performs variable selection. LASSO regression was performed using the approach found in Equation (5):

(5) $$\overset{N}{\underset{i=1}{\sum }}{\left(Y_{i}-\underset{j}{\sum }X_{ij}\beta_{j}\right)}^{2}+\alpha \overset{k}{\underset{j=1}{\sum }}\left|\beta_{j}\right|$$

where $X_{ij}$ is an independent variable matrix, and $Y_{i}$ is dependent variable when i = 1,2,…., N and j = 1,2,…..,k, N is the number of observations, k is the length of the vector, $\beta_{j}$ is regression coefficients, and α is a nonnegative regularisation parameter that is used to control penalising term $\alpha \overset{N}{\underset{j=1}{\sum }}\left|\beta_{j}\right|$. In order to optimise LASSO regression, the optimal α value needs to estimate by iteratively testing different α values with a cross-validation approach. The same cross-validation approach described in Section 2.5.1 for the PLSR model was used to estimate the optimal α values for LASSO as well.

#### 2.5.3. Model Evaluation

The predictive ability of a developed model is evaluated by the coefficients of determination (R^2^) and RMSE of the training and testing dataset [24,32,34]. The ratio of performance to deviation (RPD) may also be used to further assess the reliability of the predictions [34,52,55]. The study in [55] suggested that the predictive ability of a model could be considered ‘good’ when RPD values exceed 1.4. The R^2^, RMSE, and RPD are calculated using the following equations:

(6) $$R_{T/V}^{2}=1-\frac{\sum {\left(y_{i,p}-y_{i,m}\right)}^{2}}{\sum {\left(y_{i,p}-\;\bar{y}\right)}^{2}}$$

(7) $${\mathrm{RMSE}}_{T/V}=\sqrt{\frac{1}{m}\overset{m}{\underset{i=1}{\sum }}{\left(y_{i,p}-y_{i,m}\right)}^{2}}$$

(8) $${\mathrm{RPD}}_{T/V}=\frac{{\mathrm{SD}}_{T/V}}{{\mathrm{RMSE}}_{T/V}}$$

where y_p_ is the predicted value, y_m_ is the main value for the ith sample, m is the number of samples, $\;\bar{y}$ is the average of the actual loads of m samples, and SD is the standard deviation of the predicted values in the training (T) and testing (V) set.

The schematic diagram of the overall data processing, analysis, and model development is shown in Figure 4.

In addition, the limit of detection (LOD) was calculated for the best-performing models, following the formula provided in Equation (9). This value represented the minimum ratio of gingerol-to-shogaol which could be detected above the signal-to-noise ratio of the hyperspectral model.

(9) $$\mathrm{LOD}=\frac{3\times \mathrm{RMSE}\;\mathrm{of}\;\mathrm{the}\;\mathrm{calibration}}{\mathrm{Slope}\;\mathrm{of}\;\mathrm{the}\;\mathrm{calibration}}$$

### 2.6. Wavelength Selection Method

In multivariate data analysis, variable selection may be a helpful step to improve PLSR model performance. The PLSR regression coefficients (called β-coefficients) and the variable importance in the projection (VIP) are commonly used as variable selection methods. This study investigated both selection methods for PLSR only, as LASSO regression already performs variable selection while shrinking the coefficients.

In this study, informative wavelengths were selected using the PLSR regression β-coefficients of each variable. As demonstrated in [56], wavelengths with large positive or negative β-coefficients play a vital role and carry useful information in the PLSR model. The procedure used in [52,57] was followed to select the critical wavelengths. Wavelengths with an absolute β-coefficient value less than the standard deviation of all β-coefficients were discarded and the remaining wavelengths were retained.

VIP is another method often used for providing valuable insight into the most effective spectral regions by finding informative wavelengths [29,37,58,59]. The VIP method selects variables by first calculating the VIP score–an accumulation of the importance of each variable to each component in the PLSR model. All variables with VIP scores below a certain threshold are then excluded. In this study, the threshold value was set to 1, based on the recommendations of previous studies [37,59].

## 3. Results and Discussion

The average spectrum of all the pixels inside the ROI of each dried ginger sample was calculated before pre-processing the spectrum data. The pre-processing methods, including SNV, MF5, MF11, 1D-SG, 2D-SG, and MSC, were applied to the averaged spectra. The PLSR and LASSO models were developed based on the original averaged spectra, and their corresponding pre-processed spectra. Before selecting the optimum wavelengths, a total of 16 predictive models (8 PLSR and 8 LASSO models) were built based on the different pre-processing methods used on the HSI dataset. Table S1 in the supporting information file shows the main statistical parameters used to evaluate the performance of the PLSR and LASSP models developed in this study, including latent variables (LVs), α values, R^2^, RMSE, and RPD.

**Figure 5:**
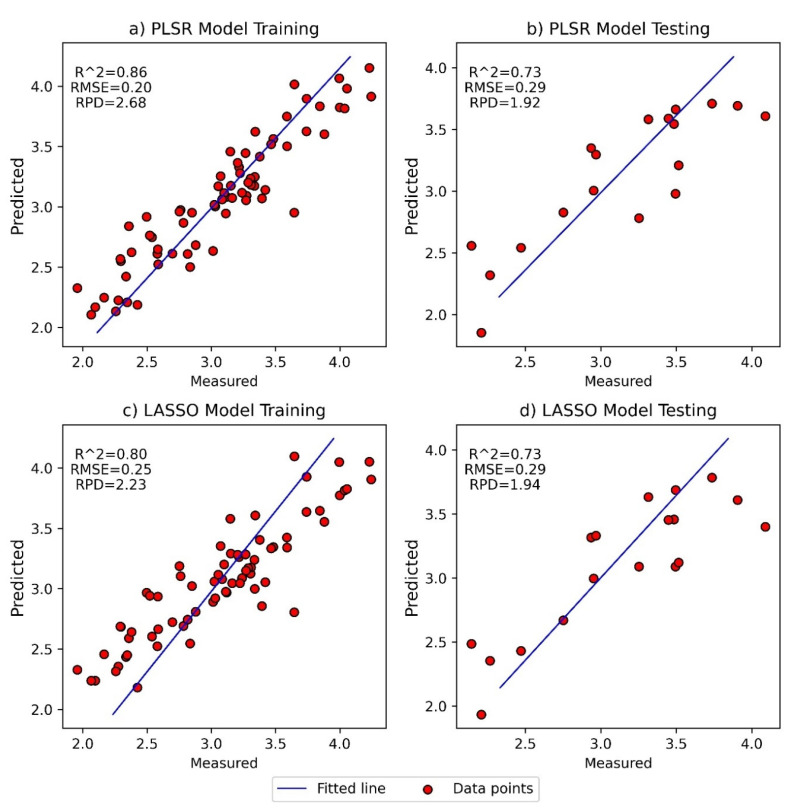
Measured vs. predicted ratio of 6-gingerol to 6-shogaol for the training and test datasets for best fit model from PLSR (**a**,**b**) and LASSO (**c**,**d**) regressions.

The performance of the predictive models was evaluated based on the R^2^ value between the predicted and measured ratios of 6-gingerol to 6-shogaol (RMSE and RPD respectively). A good model should have a high R^2^, low RMSE and a high RPD (see text S1 in the supporting information file for more details). All models developed without and with pre-processing methods for PLSR and LASSO show acceptable performance (R^2^ > 0.5, RMSE < 0.4, and RPD > 1.4) for both the training and testing dataset (Table S1). However, the best performing models using PLSR (R^2^ ≥ 0.73, RMSE ≤ 0.29, and RPD ≥ 1.92) and LASSO (R^2^ ≥ 0.73, RMSE ≤ 0.29, and RPD ≥ 1.94) were found when applying MSC and 2D-SG pre-processing, respectively. Application of these models to the test set indicated that hyperspectral imaging could predict the ratio of 6-gingerol to 6-shogaol with acceptable accuracy (Figure 5). For the training set, the calculated limits of detection were gingerol-to-shogaol ratios of 0.6 and 0.9, for the PLSR and LASSO models, respectively.

**Figure 6:**
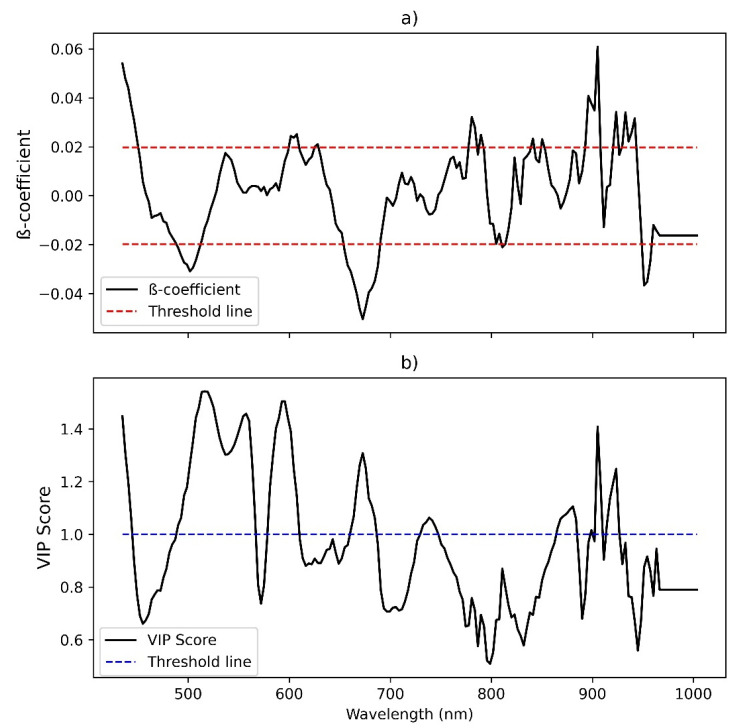
(**a**) β-coefficient for PLSR model III presented in Table S2 (The horizontal red dash lines are threshold lines based on the standard deviation for optimum wavelength selection) and (**b**) VIP score for PLSR model III presented in Table S3 (The horizontal blue dash lines are threshold lines based on the recommended VIP score from the previous study).

Subsequently, the selection of important wavelengths was performed to determine if this improved the prediction accuracy of the models. A total of 16 models were developed using the β-coefficient and VIP wavelength selection methods (see Tables S2 and S3 in the Supplementary File). When the β-coefficient method was performed, four of the eight models were poor (RPD < 1.4), and only two models (RPD ≥ 1.4) showed good agreement when the VIP method was applied. The best model for both methods was found when using the 2D-SG pre-processing technique (R^2^ ≥ 0.71, RMSE ≤ 0.30, and RPD ≥ 1.87 for PLSR β-coefficient, and R^2^ ≥ 0.72, RMSE ≤ 0.29, and RPD ≥ 1.90 for PLSR VIP). For the PLSR models, the number of wavelengths was successfully reduced to 53 and 71, using the β-coefficient and VIP variable selection methods, respectively (Figure 6).

**Figure 7:**
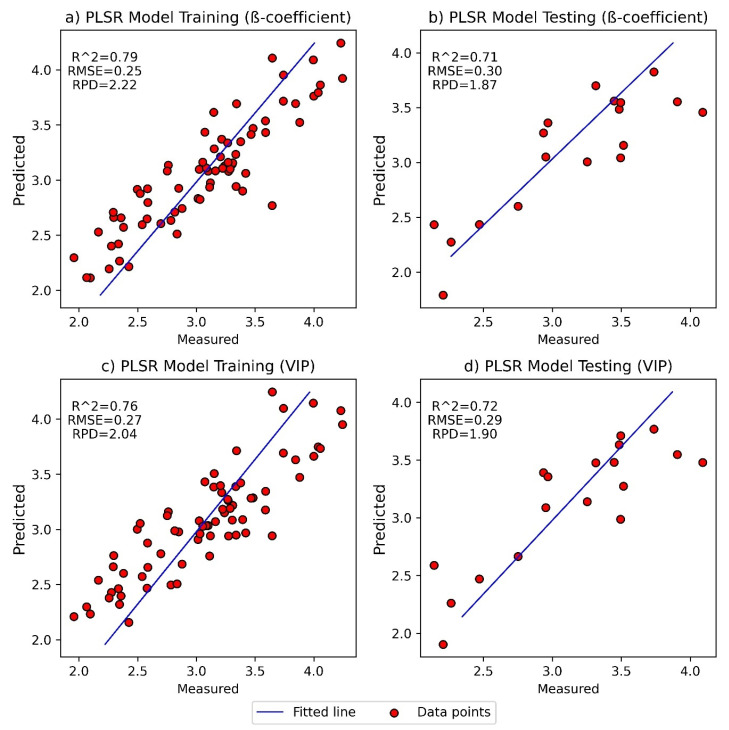
Measured vs. predicted values of the training and test datasets for PLSR model III are presented in Table S2 (**a**,**b**) and Table S3 (**c**,**d**) after selecting optimum wavelengths using β-coefficient and VIP score, respectively.

These two models showed acceptable performance after optimum wavelength selection (Figure 7), but their accuracy was lower compared to other models which did not apply optimum wavelength selection (shown in Figure 5). The calculated limits of detection for the VIP and β-coefficient models were gingerol-to-shogaol ratios of 0.8 and 1.0, respectively. These were moderately higher compared to the LODs found for the regular PLSR and LASSO models.

To the best of the authors’ knowledge, this is the first application of a HSI system to predict the pungent constituents in dried ginger. The use of larger datasets during training, including different ginger varieties, could potentially improve these results. However, the preliminary results of this study suggest that with further refinement, HSI combined with chemometric data processing may be used to estimate the concentrations of desirable pungent constituents in dried ginger powder, which could be greatly beneficial for quality assurance purposes. As 6-shogaol is approximately twice as pungent as 6-gingerol [11], dried ginger samples with a lower gingerol:shogaol ratio would be expected to have a more pungent flavour. Conversely, those with a higher gingerol:shogaol ratio would have a milder flavour. Rapid quantification of this attribute could be beneficial in certain manufacturing processes using processed ginger. Furthermore, ginger samples containing higher shogaol levels may also have greater health benefits [12]; thus, manufacturers could market products with a lower gingerol:shogaol ratio as potentially being more beneficial for the consumer.

Another application of HSI for ginger quality analysis could be for monitoring the extent of drying processes. Gingerols are converted to shogaols during the drying process through an elimination dehydration reaction involving the hydroxyl group on the alkyl chain [60]; thus, the gingerol:shogaol ratio. This could be combined with the assessment of the moisture contents through HSI, as previously reported [35,36,37].

Recent work has found that older, lower-quality samples of dried ginger showed significantly lower gingerol:shogaol ratios compared to fresher, higher-quality ginger [10,39,40]. Although this suggests that the gingerol:shogaol ratio may be a key measure of ginger quality, there are also other compounds that contribute to the perceived flavour and hence the quality of ginger [60]. Whilst it should be cautioned that further investigations may be required to demonstrate the relative contribution of the gingerol:shogaol ratio to overall ginger quality, the prospect of rapid and cheap quantification of the gingerol:shogaol ratio in ginger via hyperspectral imaging shows promise for improving quality assurance processes in the ginger processing industry.

## 4. Conclusions

This study highlighted the capability of hyperspectral imaging coupled with multivariate analysis for the real-time determination of the 6-gingerol:6-shogaol ratio in dried ginger powder. A total of 32 regression models were created, based on different spectral pre-treatment techniques and two multivariate regression methods. The results succeeded in demonstrating that it is possible to predict the 6-gingerol:6-shogaol ratio using PLSR and LASSO with acceptable performance metrics (R^2^ ≥ 0.73, RMSE ≤ 0.29 and RPD ≥ 1.92). Compared to PLSR, the use of the LASSO model improved the RPD score by 0.02, for the best-performing models obtained from each approach. This indicates that the LASSO model may be used as a substitute for PLSR when conducting the spectroscopic analysis of horticultural food products.

## Data Availability

The HPLC and HSI data are available upon request from the authors.

## Supplementary Materials

The following supporting information can be downloaded at: https://www.mdpi.com/article/10.3390/foods11050649/s1, Figure S1: Image of a ginger powder sample with the region of interest (ROI), taken by the Specim IQ hyperspectral VS-NIR camera; Table S1: Considering full wavelength for PLSR and LASSO; Table S2: Optimum wavelength selection from full wavelength based on Beta-coefficient; Table S3: Optimum wavelength selection from full wavelength based on VIP.
