# Supplementary material for: A Rapid Non-Destructive Hyperspectral Imaging Data Model for the Prediction of Pungent Constituents in Dried Ginger
Source: Foods. 2022 Feb 23;11(5):649. doi: 10.3390/foods11050649 (PMC8909893; doi:10.3390/foods11050649)
Supplement: Supplementary file 1 [file foods-11-00649-s001.zip › foods-1543580-supplementary.pdf]

"Foods"

Supporting Information for

A rapid non-destructive hyperspectral imaging data model for the prediction of pungent constituents in dried ginger

Nahidul Hoque Samrat[1,2], Joel B. Johnson[1,2], Simon White[1,2], Mani Naiker[1,2], Philip Brown[1,2]

[1] School of Health, Medical and Applied Sciences, Central Queensland University, Queensland, Australia.

[2] Institute for Future Farming Systems, Central Queensland University, Queensland, Australia.

Contents of this file

Figure S1

Text S1

Table S1-S3

Introduction

This supporting information file contains the supplementary figure, text and tables as referenced in the main article.

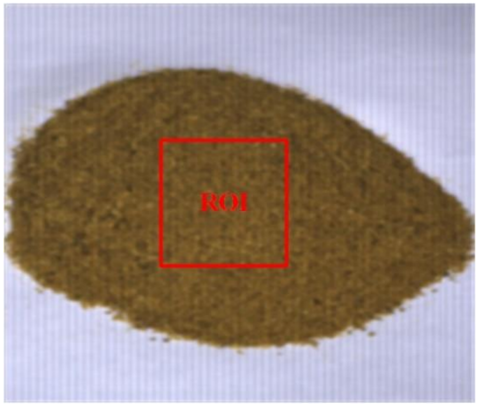

Figure S1. Image of a ginger powder sample with the region of interest (ROI), taken by the Specim IQ hyperspectral VS-NIR camera.

S1: Model evaluation and comparison

The model's accuracy was evaluated based on the coefficient of determination (R^2), root means square error (RMSE), and the ratio of performance to deviation (RPD) values. The model fitting are divided into three categories: poor, good, and best, based on the RPD values. The models that provided RPD < 1.4 for the predictions were considered as "poor", those with 1.4 < RPD <2 was considered as "good" [1-3]. Best-fit models were selected based on having the highest R^2 and RPD, and the lowest RMSE in the training and test sets.

Model categories	Color code
Poor fit	
Good fit	
Best fit	

Table S1: Considering full wavelength for PLSR and LASSO

Model	Preprocessing technique	PLSR LV	LASSO alpha value	Wavelength	PLSR						LASSO					
					Training			Test			Training			Test		
					R2	RMSE	RPD	R2	RMSE	RPD	R2	RMSE	RPD	R2	RMSE	RPD
I	None	13	0.116e-3	204	0.83	0.22	2.46	0.70	0.31	1.82	0.74	0.28	1.97	0.68	0.32	1.76
II	1D-SG	10	0.181e-3	204	0.79	025	2.20	0.61	0.35	1.62	0.86	0.20	2.74	0.55	0.37	1.50
III	2D-SG	9	0.943e-3	204	0.78	0.26	2.13	0.70	0.30	1.85	0.80	0.25	2.23	0.73	0.29	1.94
IV	SNV	13	0.413e-3	204	0.89	0.18	3.07	0.62	0.35	1.62	0.79	0.25	2.18	0.70	0.31	1.82
V	MSC	12	0.364e-3	204	0.86	0.20	2.68	0.73	0.29	1.92	0.78	0.26	2.14	0.70	0.31	1.83
VI	MF5	13	9.013e-5	204	0.79	0.25	2.23	0.70	0.31	1.82	0.71	0.30	1.86	0.66	0.32	1.73
VII	MF9	13	7.938e-5	204	0.78	0.25	2.16	0.70	0.31	1.83	0.70	0.30	1.84	0.70	0.30	1.84
VIII	MSC+SNV	13	0.413e-3	204	0.89	0.18	3.07	0.62	0.35	1.62	0.79	0.25	2.18	0.70	0.31	1.82

Lantent variable(LV); Partial least squares regression(PLSR);Least absolute shrinkage and selection operator (LASSO)

Table S2: Optimum wavelength selection from full wavelength based on Beta-coefficient

Model	Preprocessing technique	PLSR LV	Wavelength	PLSR					
				Training			Test		
				R2	RMSE	RPD	R2	RMSE	RPD
I	None	13	59	0.86	0.20	2.67	0.56	0.37	1.51
II	1D-SG	10	51	0.70	0.30	1.82	0.35	0.45	1.24
III	2D-SG	9	53	0.79	0.25	2.22	0.71	0.30	1.87
IV	SNV	13	55	0.91	0.16	3.46	0.37	0.45	1.26
V	MSC	12	59	0.89	0.18	3.01	0.49	0.40	1.40
VI	MF5	13	61	0.80	0.24	2.26	0.48	0.40	1.39
VII	MF9	13	58	0.77	0.26	2.10	0.69	0.31	1.80
VIII	MSC+SNV	13	55	0.91	0.16	3.46	0.37	0.45	1.26

Table S3: Optimum wavelength selection from full wavelength based on VIP

Model	Preprocessing technique	PLSR LV	Wavelength	PLSR					
				Training			Test		
				R2	RMSE	RPD	R2	RMSE	RPD
I	None	13	51	0.80	0.24	2.24	0.37	0.45	1.26
II	1D-SG	10	84	0.72	0.29	1.91	0.36	0.45	1.25
III	2D-SG	9	71	0.76	0.27	2.04	0.72	0.29	1.90
IV	SNV	13	70	0.79	0.25	2.20	0.17	0.51	1.10
V	MSC	12	69	0.83	0.22	2.49	0.69	0.31	1.82
VI	MF5	13	49	0.71	0.29	1.88	0.47	0.41	1.38
VII	MF9	13	48	0.68	0.31	1.78	0.44	0.42	1.34
VIII	MSC+SNV	13	70	0.79	0.25	2.20	0.17	0.51	1.10

Reference

[1] S. H. Bai et al., "A non-destructive determination of peroxide values, total nitrogen and mineral nutrients in an edible tree nut using hyperspectral imaging," Computers and Electronics in Agriculture, vol. 151, pp. 492-500, 2018.

[2] I. Tahmasbian, N. K. Morgan, S. Hosseini Bai, M. W. Dunlop, and A. F. Moss, "Comparison of Hyperspectral Imaging and Near-Infrared Spectroscopy to Determine Nitrogen and Carbon Concentrations in Wheat," Remote Sensing, vol. 13, no. 6, p. 1128, 2021.

[3] I. Tahmasbian, H. M. Wallace, T. Gama, and S. H. Bai, "An automated non-destructive prediction of peroxide value and free fatty acid level in mixed nut samples," LWT, vol. 143, p. 110893, 2021.
